# Coordinate regulation between expression levels of telomere-binding proteins and telomere length in breast carcinomas

**DOI:** 10.1002/cam4.14

**Published:** 2012-07-24

**Authors:** Kimberly S Butler, William C Hines, Christopher M Heaphy, Jeffrey K Griffith

**Affiliations:** Department of Biochemistry and Molecular Biology, University of New Mexico School of MedicineAlbuquerque, New Mexico, 87131

**Keywords:** Breast cancer, telomere maintenance, telomere-binding proteins

## Abstract

Telomere dysregulation occurs in both the in situ and invasive stages of many carcinomas, including breast. Knockout experiments have identified several telomere-associated proteins required for proper telomere function and maintenance, including telomere repeat-binding factor 1 and 2 (TRF1 and TRF2), protection of telomeres (POT1), and TRF1-interacting nuclear factor 2 (TIN2). Using telomere content assays and quantitative reverse transcription-polymerase chain reaction (RT-PCR), we examined the relationship between telomere length and the mRNA levels of telomere-associated proteins in breast tumors. The levels of TRF2, TRF1, TIN2, and POT1 mRNA, but not telomerase reverse transcriptase (TERT) RNA, are inversely correlated with telomere content in breast tumors. Significant associations were identified between the mRNA levels of TRF1, TIN2, and POT1; however, there were no significant associations with the mRNA levels of TRF2 or TERT. These associations suggest that a complex transcriptional program coordinately regulates the expression of these mRNAs. We examined the promoter regions of the telomere-associated proteins to identify transcription factors consistent with the observed patterns of presumed coordinate expression. We demonstrated in human breast cancer cell lines that expressions of TRF1, TIN2, and POT1 are upregulated by dexamethasone, suggesting activation of the glucocorticoid receptor, whereas TERT, TRF2, TRF1, TIN2, and POT1 are upregulated by tumor necrosis factor-*α* (TNF-*α*), suggesting activation of the nuclear factor kappa B transcription factor. These findings link telomere content in breast tumors to the coordinate expression of several telomere-associated proteins previously shown to be negative regulators of telomere length in cell lines. The results further suggest a possible link between the expressions of the telomere-associated proteins and mediators of stress and inflammation.

Telomere content assays and quantitative RT-PCR demonstrate that the levels of TRF2, TRF1, TIN2, and POT1 mRNA, but not telomerase reverse transcriptase (TERT) RNA, are inversely correlated with telomere content in breast tumors. Within human breast cancer cell lines, expressions of TRF1, TIN2, and POT1 are upregulated by dexamethasone, suggesting activation of the glucocorticoid receptor, whereas TERT, TRF2, TRF1, TIN2, and POT1 are upregulated by TNF-*α*, suggesting activation of the NFκB transcription factor. These findings link telomere content in breast tumors to the expression of several telomere-associated proteins previously shown to be negative regulators of telomere length in cell lines and suggest a link between the expressions of the telomere-associated proteins and mediators of stress and inflammation.

## Introduction

Telomeres are specialized protein–nucleic acid structures that stabilize the ends of chromosomes, thereby preventing activation of DNA damage pathways, exonucleolytic degradation, and end-to-end fusion of chromosomes [1, 2]. Several telomere-associated proteins are necessary for proper telomere function and maintenance and together form a complex called shelterin [3]. Telomere repeat-binding factor 1 and 2 (TRF1 and TRF2) bind telomere DNA duplexes directly and nucleate the formation of multiprotein complexes. The TRF2 and TRF1 complexes have different roles in telomere maintenance [4]. The TRF2 complex is necessary for the formation of the T-loop, the structure that caps and protects the ends of the telomeres. Loss of TRF2 has little effect on double-strand telomere DNA, but results in the loss of the single-strand TTAGGG repeat at the end of the telomere. The resulting structure resembles a double-strand DNA break and can induce apoptosis or senescence, and create a substrate for telomere–telomere fusions [5, 3]. In contrast, the TRF1 complex regulates telomere elongation [6]. The TRF1 complex includes adrenocortical dysplasia homolog, TRF1-interacting nuclear factor 2 (TIN2), and protection of telomeres 1 (POT1). POT1 binds single-strand TTAGGG repeats at the 3′ overhang, preventing binding of telomerase, the specialized reverse transcriptase that adds telomere DNA to the ends of chromosomes [7, 8]. POT1 is bound to TRF1 by TIN2. Thus, reducing the levels of TRF1, TIN2, or POT1 can decrease POT1 binding, expose the single-strand TTAGGG repeats at the 3′ overhang, and increase access to telomerase binding and thereby increase telomere length. Consistent with this scheme, experiments in genetically manipulated cells demonstrate that TRF2, TRF1, TIN2, and POT1 are all negative regulators of telomere length [6, 9–11].

Telomere dysregulation is a common occurrence in breast and other cancers [12–14]. For example, in a study of 140 human breast tumors, 50% had telomere lengths that were longer or shorter than telomere lengths in >95% of normal tissues [13, 15]. The quantitative relationships between telomere length and the expression of telomere-associated proteins are poorly understood. For example, although telomerase is expressed in >85% of human cancers, including breast cancers, the levels of telomerase reverse transcriptase (TERT) mRNA are not correlated with telomere length and can differ more than 700-fold in human breast cancer tissues [16]. Similarly, it is not known how expressions of TRF2, TRF1, TIN2, and POT1 in tumors are regulated or correlated with telomere length. Therefore, one objective of the investigation was to compare the expression levels of each mRNA to telomere length and to one another in a set of human breast tumor tissues.

We demonstrate in this study that the levels of TRF2, TRF1, TIN2, and POT1 mRNA, but not TERT mRNA, are inversely correlated with telomere content (a surrogate for telomere length) in human breast tumor tissues. In addition, we observed significant associations between the mRNA levels of TRF1, TIN2, and POT1, suggesting coordinate regulation, which was supported by scans of the promoter regions of the telomere-associated proteins. Therefore, the second, subsequent objective of the investigation was to test the predication that the expressions of TRF1, TIN2, and POT1 could be regulated coordinately in human breast tissue cell lines by agents chosen to activate the identified transcription factors. We then demonstrated that TRF1, TIN2, and POT1, but not TRF2 or TERT genes, are upregulated by dexamethasone, suggesting activation of the glucocorticoid receptor, while TERT, TRF2, TRF1, TIN2, and POT1 genes are upregulated by tumor necrosis factor-*α* (TNF-*α*), suggesting activation of the nuclear factor kappa B (NFκB) transcription factor. These findings suggest a link between telomere length in breast tumors and potential regulation of several telomere-associated proteins, which have previously been shown to be negative regulators of telomere length. These findings could also link expression of the telomere-associated proteins to physiological mediators of stress and inflammation that have previously been associated with breast cancer progression [17–20].

## Methods

### Breast tumor tissue

Thirty-six specimens of frozen breast tumor tissue derived from anonymous surgical remnant tissues of patients undergoing radical mastectomy were provided by the University of New Mexico Human Tissue Repository. In most cases, histopathological grade, tumor size, lymph node status, estrogen receptor status, and age of patient were also available and are detailed in Table 1. Sections of the collected tumors were examined by a pathologist to confirm that samples used for analysis contained predominantly cancerous tissue. RNA and DNA were extracted from bulk tissues without enrichment of any particular cell type.

**Table 1:** Characteristics of breast tumors

Age of patient (years)	33–89
Tumor size (cm)	<1–14
Tumor grade	
Grade 1	2
Grade 2	13
Grade 3	21
Node status	
Positive nodes	14
Negative nodes	18
Node status unknown	4
ER status	
ER negative	11
ER positive	20
ER status unknown	5

### Propagation of cell lines and drug treatments

Three human breast cell lines were chosen to represent varying levels of aggressiveness; breast adenocarcinoma cell lines MDA MB 231 and MCF7 and fibrocystic disease cell line MCF 10-2A (ATCC, Manassas, VA) were grown in media consisting of dulbecco's modified Eagle's medium (DMEM):F12 media (ATCC) supplemented with 0.01 mg/mL bovine insulin and 10% fetal bovine serum. HEK 293 cells (ATCC) were grown in DMEM media supplemented with 10% fetal bovine serum. All cell lines were grown at 37°C and 5% CO_2_. HEK 293 and MDA MB 231 cells were treated for 24 h with PMA (phorbol-12-myristate-13-acetate, 4 nmol/L), ionomycin (50 nmol/L), dexamethasone (300 ng/mL), or TNF-*α* (10 ng/mL) in quadruplicate. PMA, ionomycin, and dexamethasone were purchased from Sigma (Saint Louis, MO). TNF-*α* was purchased from R&D systems (Minneapolis, MN). For long-term treatment, cell lines were incubated with TNF-*α* (10 ng/mL), dexamethasone (300 ng/mL), or media for 28 days in quadruplicate. Medium and drugs were refreshed every 48 h. Cell pellets were collected by manual removal of cells from culture flasks followed by centrifugation, washed in phosphate buffered saline, and then snap frozen in liquid nitrogen for subsequent RNA and DNA isolation.

### RNA and DNA extraction

RNA and DNA were isolated, in parallel, from either 20 ten-micron-thick sections of frozen tumor specimens or frozen cell pellets using silica-based spin-column extraction kits (RNeasy/DNeasy mini kits; Qiagen, Valencia, CA). RNA extraction was performed according to the manufacturer's suggested protocols. However, the first wash eluate, containing the genomic DNA, was saved in a microcentrifuge tube and reserved for DNA purification. The total RNA was eluted in 50 *μ*L of RNase-free water and treated with DNase I (DNA-free; Ambion, Austin, TX). The integrity of the RNA was evaluated by visualizing the 28 s and 18 s bands after electrophoresis on a 1% agarose gel with Gelstar Nucleic Acid Stain (BioWhittaker, Rockland, ME), as well as by real-time reverse transcription-polymerase chain reaction (RT-PCR) of TATA-binding protein (TBP), transcript which serves as an internal control. The concentration of RNA was measured spectrophotometrically. DNA was quantified fluorometrically with PicoGreen (Molecular BioProbes, Eugene, OR). Placenta and HeLa cell DNA, which serve as controls for the telomere DNA content (TC) assay (described below), were purified as described [21].

### Quantitative real-time RT-PCR

TERT and TBP mRNA levels were quantified using the Taqman® real-time PCR assay [22] and dual labeled probes, whereas TRF1, TRF2, TIN2, and POT1 mRNA levels were quantified using the Sybr green real-time PCR assay (Invitrogen, Carlsbad, CA). The sequences of the primers can be found in Table 2. All PCR reactions were performed in quadruplicate using the following cycling parameters: 50°C hold × 2 min, 95°C hold × 10 min, 50 cycles: (95°C × 15 sec, 60°C × 1 min). The levels of TERT, TRF1, TRF2, TIN2, POT1, and TBP mRNAs were calculated from a standard curve generated by amplification of known amounts of the TERT, TRF1, TRF2, TIN2, POT1, and TBP standards (plasmid DNA containing the respective target sequence) using the Gene Amp® 7000 Sequence Detection System (Applied Biosystems, Foster City, CA). Baseline fluorescence was determined in each tube during PCR cycles 6–15. The fluorescent threshold level was set as recommended by the manufacturer. The normalized TERT, TRF1, TRF2, TIN2, and POT1 mRNA levels were calculated by dividing the mean copy number of the mRNA of interest by the mean copy number of TBP and multiplying by 100.

**Table 2:** Quantitative PCR primers and Taqman probes for telomere-associated proteins

PCR mRNA target	Forward primer	Reverse primer
TERT RT-PCR assay [22]	5′-CGG AAG AGT GTC TGG AGC AA-3′	5′-GGA TGA AGC GGA GTC TGG A-3′
TRF1	5′-GAC ACT GGG GAG GTA GGG T-3′	5′-GCT AAC AAA CCT GCC CAT G-3′
TRF2	5′-TCA ATC GCT GGG TGC TCA A-3′	5′-GTA CCG GCT ACC CCG AAA G-3′
TIN2	5′-CCT GGC ACA CAT CTT CCT C-3′	5′-GGC CGA CGA AGA GTT CAG T-3′
POT1	5′-CGC TTT CAC AGG CTG AAG ATT C-3′	5′-CCA AAG TTC CCT CAA ACG TCA A-3′
TBP	5′-CAC GAA CCA CGG CAC TGA TT-3′	5′-TTT TCT TGC TGC CAG TCT GGA C-3′
Taqman probes		
TERT Taqman® probe sequence	5′-6FAM-TGC TTC CGA CAG CTC CCG CAG-TAMRA	
TBP Taqman® probe sequence	5′-6FAM-TGT GCA CAG GAG CCA AGA GTG AAG A-TAMRA	

### Telomere DNA content assay

TC was measured by the slot blot assay, as previously described [23]. Briefly, DNA was denatured, neutralized, loaded, and fixed to Tropilon-Plus™ membranes (Applied Biosystems). A telomere-specific oligonucleotide end-labeled with fluorescein, (5′ TTAGGG 3′)_4_-FAM, (IDT, Coralville, IA) was hybridized to genomic DNA. Following hybridization, the blots were washed to remove nonhybridizing oligonucleotides. Telomere-specific oligonucleotides were detected by an alkaline phosphatase-conjugated antifluorescein antibody that produces light when incubated with the CDP-Star™ substrate (Applied Biosystems). Blots were exposed to Hyperfilm™ for 2–5 min (Amersham Pharmacia Biotech, Buckinghamshire, UK) and digitized. The intensity of the telomere hybridization signal was determined from the digitized images with Nucleotech Gel Expert Software 4.0 (Nucleotech, San Mateo, CA). Tumors' TC were expressed as a percentage of the average chemiluminescent signal compared to the value of the placental standard of the same mass of genomic DNA (typically 20 ng). DNA from each tumor tissue was analyzed independently three times, each in triplicate.

### Western blot analysis of TRF1 and TRF2

Protein was extracted from tumor tissues using the T-PER Tissue Protein Extraction Reagent (Pierce, Rockford, IL). Total protein concentrations for each sample were determined using a colorimetric protein assay (Protein Assay Dye Reagent Concentrate, Bio-Rad Laboratories, Hercules, CA) and comparing with a standard curve of bovine serum albumin. Proteins extracted from tumor tissues and cell lines were separated by sodium dodecyl sulphate polyacrylamide gel electrophoresis using standard protocols on 10% gel. The size of the protein was estimated from a protein size standard run in parallel with the experimental samples. Following electrophoretic separation, the proteins were transferred to a nitrocellulose membrane using a Trans-blot SD Semi-Dry Transfer Cell (Bio-Rad Laboratories, Hercules, CA). Once transferred, the membranes were washed and preblocked in 5% powdered milk in TBS-Tween. Primary antibodies to TRF1, TRF2 (Abcam, Cambridge, MA) and a control antibody to *β*-actin (Sigma) were applied overnight at 4°C. The secondary antibodies (Sigma) were conjugated to alkaline phosphatase, which produces light when incubated with the CDP-Star substrate. The blots were then exposed to hyperfilm and the intensity of the protein signal was determined from the digitized film images with Gel Expert Software 4.0. The levels of TRF1 and TRF2 were normalized to *β*-actin. Each gel contained a calibration sample, a 1:1 mixture of MCF7 and HEK 293 cell extracts (Abcam), which allowed comparison between gels.

### Statistical analysis

Simple linear regression analysis was performed using JMP IN version 5.1 statistical analysis program (SAS Institute Inc., Cary, NC) to determine the significance of the association between the variables of interest and create the graphics. General linear model regression procedure was performed using SAS software version 8.2 (SAS Institute Inc.) to calculate the significance of the association between the variables of interest as well as the correlation coefficient (*r*) and coefficient of determination (*r*^2^) values for each association.

## Results

### Telomere-associated proteins' mRNAs are correlated with telomere content in human breast tumors

Telomere-associated proteins' mRNA levels were measured in 36 human breast tumors. Telomere-associated proteins' mRNA levels were initially compared with characteristics of the tumors tissues and patients, including tumor grade, tumor size, lymph node status, estrogen receptor status, and patient age. No associations were found between the majority of the telomere-associated proteins mRNA levels and any of these tumor features. One exception was increased levels of POT1 mRNA correlated linearly with increased tumor size (*P* = 0.002, data not shown). However, given the limited number of small tumors present in this sample set (six samples below 2 cm), this relationship will require further examination with a larger set of tumors.

**Figure 1:**
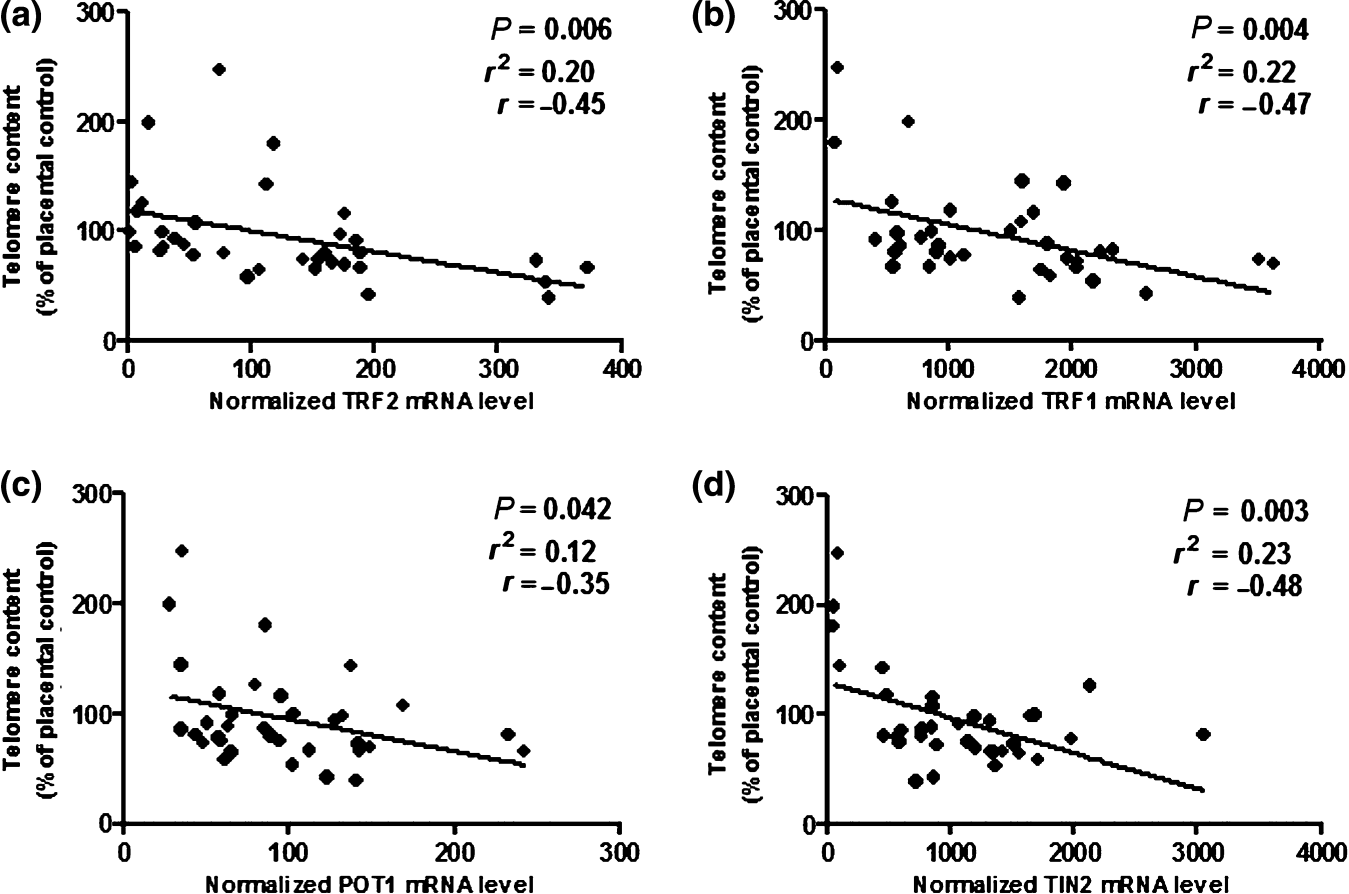
Relationships between levels of mRNAs for telomere-associated proteins and telomere DNA content (TC). TC was compared with mRNA levels of TRF1, TRF2, POT1, and TIN2 assessed by quantitative RT-PCR in 36 human breast tumors. Lines on each graph were fit using simple linear regression, and statistical analysis was done to determine the significance (*P*-value), coefficient of determination (*r*^2^), and the correlation coefficient (*r*). (a) TRF2 mRNA level compared with TC, this graph shows a significant increase in TRF2 mRNA levels with decreased TC (*P* = 0.006). (b) TRF1 mRNA level compared with TC, this graph shows a significant increase in TRF1 mRNA levels with decreased TC (*P* = 0.004). (c) POT1 mRNA level compared with TC, this graph shows a significant increase in TRF1 mRNA levels with decreased TC (*P* = 0.042). (d) TIN2 mRNA level compared with TC, this graph shows a significant increase in TRF1 mRNA levels with decreased TC (*P* = 0.003).

Mean telomere DNA lengths, measured as TC, were measured in 36 human breast tumors and compared with the telomere-associated proteins' mRNA levels. As shown in Figure 1, linear regression analysis revealed significant, negative, linear associations between each of these mRNAs and TC (*P* = 0.006, 0.004, 0.003, and 0.042, respectively). In each instance, there was a significant *P*-value but a weak coefficient of determination (*r*^2^). The *P*-values indicate there is a statistically significant association between levels of the telomere-associated proteins' mRNAs and TC, while the weak coefficient of determination indicates a single variable alone cannot predict TC entirely. In contrast, there was no significant association between TERT mRNA levels and TC (*P* = 0.523, data not shown), which confirms previous studies [24, 25]. These results, obtained in human breast tumors, are consistent with the complex model based on genetically modified cell lines, in which TRF1, TRF2, TIN2, and POT1 are each negative regulators of telomere length [10, 11, 26, 27].

**Figure 2:**
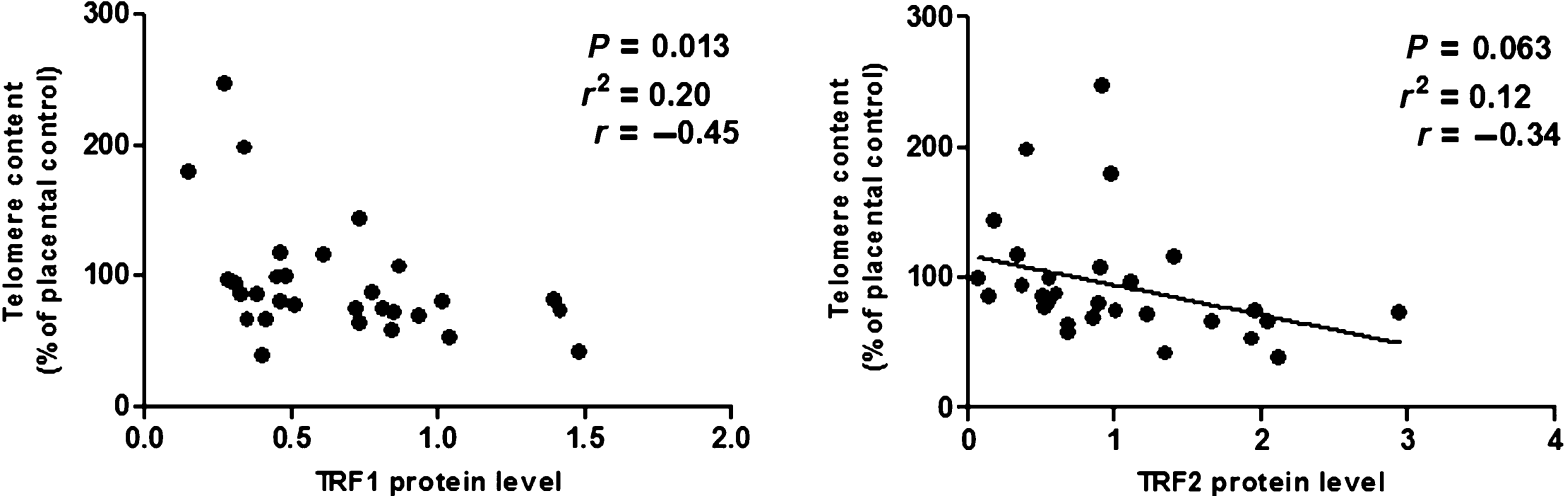
Relationship between levels of TRF1 and TRF2 protein and telomere DNA content (TC). TC was compared with protein levels of TRF1 and TRF2 measured by Western blot in 36 human breast tumors. Lines on each graph were fit using simple linear regression, and statistical analysis was done to determine the significance (*P*-value), coefficient of determination (*r*^2^), and the correlation coefficient (*r*). (a) TRF1 protein level compared with TC, this graph shows a significant increase in TRF1 protein levels with decreased TC (*P* = 0.013). (b) TRF2 protein level compared with TC, this graph shows the trend for increased TRF2 protein levels with decreased TC (*P* = 0.063).

To examine further the relationship between TC and the telomere-associated proteins in tumor tissues, we compared the protein levels of TRF1 and TRF2 to TC (Fig. 2). TRF1 and TRF2 were chosen because their mRNA levels are strongly correlated with mean telomere content and they interact with telomere DNA directly and independently [4]. TIN2 was not chosen because it interacts with the DNA indirectly through TRF1. Similarly, POT1 was not chosen because the interaction of POT1 with single stranded telomere DNA is affected by TRF1 and is therefore not independent of the other telomere-associated proteins [4]. TRF1 and TRF2 protein levels also were associated negatively with TC (*P* = 0.013 and 0.063, respectively) and strongly associated with their corresponding mRNA levels (data not shown). The association of the protein levels with TC supports the conclusion that the mRNA levels of the telomere-associated proteins are reflective of the protein levels.

### Coordinate expression of telomere-associated proteins in human breast tumors

**Figure 3:**
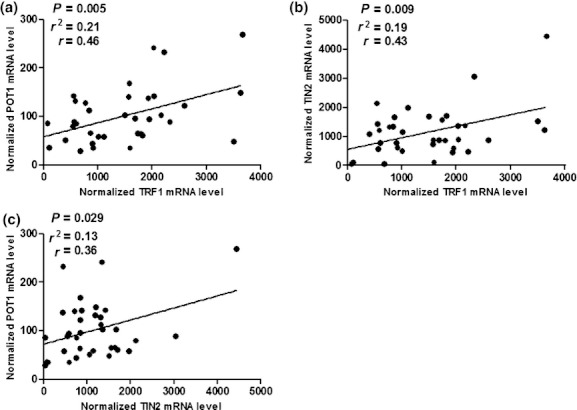
Relationship between levels of TRF1 and TRF1-associated protein mRNAs. The mRNA levels of TRF1, POT1, and TIN2 were assessed by quantitative RT-PCR in 36 human breast tumors. Lines on each graph were fit using simple linear regression, and statistical analysis was done to determine the significance (*P*-value), coefficient of determination (*r*^2^), and the correlation coefficient (*r*). (a) POT1 mRNA levels were compared with TRF1 mRNA levels, showing a significant positive association (*P* = 0.005). (b) TIN2 mRNA levels were compared with TRF1 mRNA levels, showing a significant positive association (*P* = 0.009). (c) POT1 mRNA levels were compared with TIN2 mRNA levels, showing a significant positive association (*P* = 0.029).

**Figure 4:**
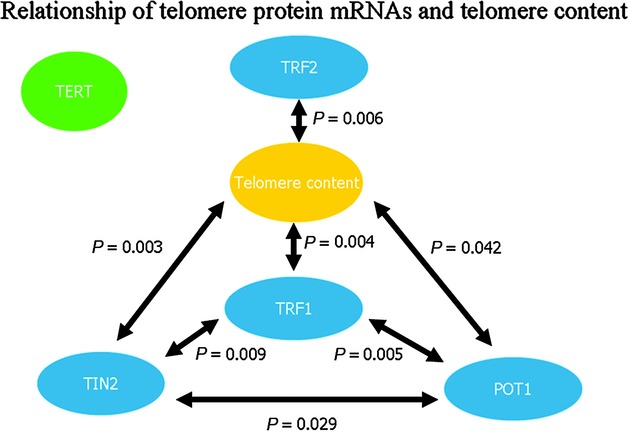
Schematic representation of the relationships between the telomere-associated protein mRNA levels and telomere content. The relationships between the mRNA levels of the telomere-associated proteins, as well as telomere content, are represented. The arrows connect the two correlated mRNAs or TC and the *P*-values are shown next to the arrows. TERT mRNA levels showed no association with either TC or other telomere-associated protein mRNAs. TC is significantly associated with TRF2, TRF1, TIN2, and POT1 mRNA levels. TRF2 mRNA levels showed no correlation with any of the other mRNA levels. TRF1, TIN2, and POT1 mRNA levels all showed significant associations with one another.

The finding that the mRNA levels of TRF1, TRF2, TIN2, and POT1 were each correlated with mean telomere length suggests these mRNAs are regulated coordinately and that the levels of the mRNAs are related significantly to one another. To test this hypothesis, the mRNA levels were compared by regression. Strong pairwise associations were indicated between TRF1 and POT1 mRNAs (*P* = 0.005; Fig. 3a), TRF1 and TIN2 mRNAs (*P* = 0.009; Fig. 3b), and TIN2 and POT1 mRNAs (*P* = 0.029; Fig. 3c). In contrast, there were no significant associations between either TRF2 or TERT and any of the telomere-associated proteins mRNAs (data not shown). The complex relationships between the expression of each telomere-associated protein and telomere content and the interrelationships between the expression of telomere-associated proteins are summarized in Figure 4.

### Comparison of promoter regions of the telomere-associated proteins genes

The model represented by Figure 4 predicts that TRF1, TIN2, and POT1 contain promoter elements lacking in TRF2 and TERT. To investigate this possibility, we used the promoter element predicting program, Tfsitescan (http://www.ifti.org/cgi-bin/ifti/Tfsitescan.pl) to identify shared and unique promoter sites in the 1000 base pairs before the start site of TRF1, TRF2, TIN2, POT1, and TERT. Tfsitescan identified over 300 promoter elements in each promoter region, many of which were tissue specific or appeared in only one of the five promoter regions. Two promoter elements were identified that were common to all five telomere proteins, NFκB and AP1. Three potential promoter elements specific to TRF1, TIN2, and POT1 were also identified: NFAT, GR, C/EBP. Each of these elements has potential binding sites in the promoters of TRF1, TIN2, and POT1 that are not present in the promoters of TERT or TRF2.

**Figure 5:**
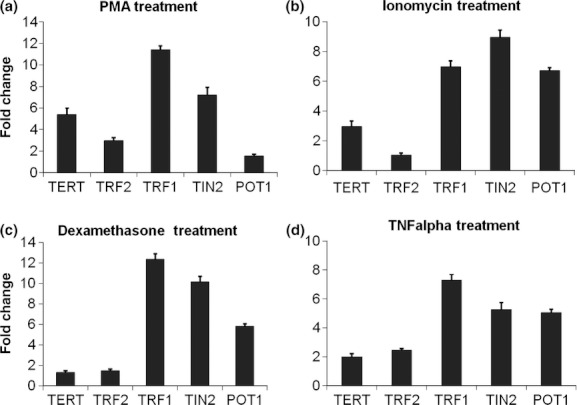
Fold change in mRNA levels of the telomere-associated proteins in response to drug treatment. MDA MB 231 cells were treated for 24 h with dexamethasone (300 nmol/L) prior to RNA extraction. Treatment was performed in quadruplicate and expression of the telomere-associated protein mRNAs is displayed as fold change compared with the untreated control for each mRNA. Error bars indicate the standard deviation of the quadruplicate treatments. (a) PMA treatment increased TERT, TRF1, TRF2, and TIN2 mRNA levels greater than 2-fold. POT1 showed a slight increase in mRNA level. (b) Ionomycin treatment increased TRF1, TERT, TIN2, and POT1 mRNA levels greater than 2-fold. TRF2 mRNA levels were unaffected. (c) Dexamethasone treatment increased TRF1, TIN2, and POT1 mRNA levels greater than 5-fold. TRF2 and TERT mRNA levels were minimally affected. (d) TNF-*α* treatment increased TRF1, TIN2, and POT1 mRNA levels greater than 5-fold. TRF2 and TERT mRNA levels were increased approximately 2-fold.

We tested the Tfsitescan predictions by treating cultured representative breast cancer cell line, MDA MB 231, for 24 h with known activators for NFκB (TNF-*α*), NFAT (PMA or ionomycin), GR (dexamethasone), and AP1 (PMA) and measuring the levels of each mRNA by qRT-PCR. The effects of C/EBP were not evaluated. As predicted, TNF-*α* increased expressions of all five proteins 2- to 7-fold, and dexamethasone selectively increased expressions of TRF1, TIN2, and POT1 6- to 12-fold compared with nontreated controls (Fig. 5). Contrary to the predictions, activation of NFAT by ionomycin increased expression of TERT 3-fold, and activation of AP1 by PMA did not increase expression of POT1. The same patterns of activation were observed in HEK 293 cells (not shown).

The coordinate increase of TERT, TRF2, TRF1, TIN2, and POT1 mRNA expressions by TNF-*α* and the selective, coordinate increase of TRF1, TIN2, and POT1 mRNA expressions by dexamethasone were repeated in MDA MB 231 cells and two additional breast cell lines, MCF7 and MCF 10-2A (Table 3). The three breast cancer cell lines were chosen to represent varying levels of aggressiveness in breast disease. MDA MB 231 and MCF7 are tumor-derived cell lines, whereas MCF 10-2A is an immortal cell line derived from a fibrocystic lesion [28].

**Table 3:** Changes in transcription levels of the telomere-associated proteins in response to 24-h dexamethasone and TNF-*α* treatments in three human breast-derived cell lines

	MDA MB 231 fold increase from untreated controls (standard deviation)	MCF7 fold increase from untreated controls (standard deviation)	MCF 10-2A fold increase from untreated controls (standard deviation)
Dexamethasone			
TRF2	1.2 (0.13)	1.0 (0.06)	1.0 (0.06)
TERT	1.0 (0.01)	1.0 (0.03)	1.1 (0.01)
TRF1	11.7 (0.81)	9.6 (0.38)	8.7 (0.63)
TIN2	10.3 (0.71)	8.4 (0.37)	8.1 (0.44)
POT1	5.1 (0.56)	6.1 (0.14)	4.8 (0.10)
TNF-*α*			
TRF2	2.1 (0.15)	2.1 (0.08)	1.5 (0.09)
TERT	1.9 (0.01)	1.5 (0.05)	1.0 (0.02)
TRF1	6.5 (0.53)	6.5 (0.19)	6.4 (0.41)
TIN2	4.8 (0.43)	4.7 (0.34)	5.4 (0.49)
POT1	4.1 (0.54)	5.1 (0.11)	5.0 (0.11)

In each cell line, maximum expressions of TRF1, TRF2, TERT, POT1, and TIN2 were achieved after 24-h exposure to TNF-*α* or dexamethasone (Table 3). Dexamethasone increased the expressions of TRF1 8.8- to 11.7-fold, POT1 4.8- to 6.1-fold, and TIN2 8.0- to 10.3-fold, depending on the cell line. Dexamethasone did not increase the expression of TRF2 or TERT in any of the three cell lines. TNF-*α* increased expression of TRF1 6.4- to 6.5-fold, POT1 4.1- to 5.1-fold, and TIN2 4.7- to 5.4-fold, depending on the cell line. TNF-*α* had a smaller effect on the expressions of TRF2 and TERT. Expression of TRF2 increased 1.5- to 2.1-fold in the three cell lines. TERT increased 2.0-fold in MDA MB 231, but only 1.5-fold in MCF7 cells, and no increase was detected in MCF 10-2A cells. No further change in expression of any of the five mRNAs occurred during four subsequent weeks of continuous exposure to physiological levels of either TNF-*α* (10 ng/mL) or dexamethasone (300 ng/mL). During this time, the growth rate of MCF7 cells treated with TNF-*α* was 95% of the untreated control, whereas TNF-*α* reduced the growth rate of MCF 10-2A and MDA MB 231 cells by 34% and 44%, respectively. Similarly, dexamethasone reduced the growth rates of all three cell lines by 35–40%. Overall, treated cells underwent 8–10 population doublings, but no reduction in telomere content was observed relative to untreated controls (data not shown).

## Discussion

Dysregulation of telomere length has important implications in cancer. Telomere attrition is a significant cause of genomic instability, and an early event in tumorigenesis [29]. Telomere length is also an independent and robust predictor of clinical outcome and disease-free survival following surgery in cancer [12, 13, 30–33], implying that telomere length reflects cellular phenotype. Several telomere-associated proteins have been implicated in telomere stability and control of telomere length (reviewed in [3, 4, 34]), suggesting that the regulation of expression of the telomere-associated proteins could be an important determinant of clinical outcome in breast cancer. This study investigated the relationship between telomere content and the expression of several telomere-associated protein mRNAs in human breast tumors.

Two principal conclusions emerge from this study. First, there is a significant, negative association between telomere content and the levels of mRNA expressions of TRF2, TRF1, TIN2, and POT1 in human breast tumors. Similar negative associations between telomere length and telomere-associated proteins have been reported previously in gastric cancer [35–37]. The coefficient of determination (*r*^2^) of the protein levels of TRF1 and TIN2 to telomere length in this prior study was virtually identical to the protein and mRNA level correlations presented here [35]. However, the coefficient of determination of protein level of TRF2 to telomere length in gastric cancers was stronger than we observed (*r*^2^ = 0.3 vs. 0.2) [35]. In contrast to our findings, these authors reported a significant, positive association between POT1 mRNA levels and telomere length [36, 37]. Their result is surprising given the coordinate expressions of TRF1, TIN2, and POT1 observed in both breast tissue and breast cell lines treated with TNF-*α* and dexamethasone. It is possible that this discrepancy represents a difference in gene expression in breast and gastric tumors. For example, a study in gastric cancer has shown that TERT protein expression is inversely correlated with telomere length [35], while we and other have found that TERT mRNA levels in breast cancers are not associated with telomere content [24, 38–40]. However, each of the telomere-associated protein mRNAs also had a weak coefficient of determination (*r*^2^). Taken together, these findings indicate that none of these factors alone predict telomere content in breast tumors. The observed results are consistent with the currently proposed model of telomere regulation, based on experiments in genetically modified cells, which has demonstrated that TRF2, TRF1, TIN2, and POT1 are each negative regulators of telomere length. The results of this study suggest that the proposed regulation determined experimentally with cell lines also exists within human breast tumors [9, 10, 41, 42].

Regression analyses suggest that the expressions of TRF1, TIN2, and POT1, all members of the TRF1 complex, are regulated coordinately, and independent of TRF2, which forms a separate complex with Rap1. As described above, the TRF1 and TRF2 complexes have different roles in telomere maintenance. This may account for our observations that (i) expression of the proteins comprising the TRF1 complex is coordinate, (ii) expression of the proteins comprising the TRF1 and TRF2 complexes are each correlated with telomere length, but (iii) expression of TRF2 is independent of expression of the proteins comprising the TRF1 complex. Therefore, we tested the prediction that expressions of TRF1, TIN2, and POT1 are regulated coordinately, and independent of TRF2.

Two potential promoter binding elements were identified that were common to all five telomere proteins and are targets for the transcription factors NFκB and AP1. TNF-*α* or PMA were added, respectively, to stimulate these transcription factors. Three potential promoter elements specific to TRF1, TIN2, and POT1 were also identified that are targets for NFAT, GR, and C/EBP transcription factors. To stimulate NFAT or GR, ionomycin or dexamethasone was added, respectively. The predicted patterns of expression were obtained after exposure to TNF-*α* and dexamethasone, although TNF-*α* had only a small effect on TRF2 and TERT. The predicted patterns of expression were partially achieved by activation of NFAT and AP1. These data provide additional support for the conclusion that the expression of the telomere-associated proteins is regulated coordinately, potentially by some of the transcription factors listed above; however, additional experimentation, including chromatin immunoprecipitation assays, will be required to definitively demonstrate which transcription factor(s) is/are responsible for upregulation of the expression of the telomere-associated proteins. Nonetheless, it is provocative that each of the transcription factors implicated by these experiments in the expression of these telomere-associated proteins has been previously linked to breast cancer progression or apoptosis [17–20, 43–45].

A second conclusion from these data are that the expressions of TERT, TRF2, TRF1, TIN2, and POT1 can be regulated by factors presumed to be at the tumor site, including mediators of stress (glucocorticoids) and inflammation (TNF-*α*). Previous publications have shown that overexpressions of TRF1, TRF2, or TIN2 result in telomere shortening [9, 41, 42], and loss of POT1 function leads to telomere elongation [10]. Similarly, our study shows that telomere content in breast tumors also is inversely associated with levels of mRNAs of these proteins. This could indicate that telomere content in tumors is influenced by the effects of bioactive compounds, such as TNF-*α*, on the expression of telomere-associated proteins. However, we also found that telomere contents in the cell lines were not reduced by continuous 4-week exposures of cultured breast cells to either TNF-*α* or dexamethasone, although each treatment resulted in 5- to 10-fold increases in TRF1, TIN2, and POT1. This apparent contradiction is likely explained by the data of van Steensel and colleagues who reported that overexpression of TRF1 shortens telomeres at a rate of only 3–11 bp/population doubling. Our 4-week exposures resulted in only 8–10 population doublings, and arguably the loss of only 25–110 bp of telomere DNA. This is only 1–2% of the telomere lengths measured in these cell lines and below the sensitivity of the TC assay. Future studies with longer exposures will be necessary to determine definitively if telomere shortening occurs in response to inflammatory factors. We also cannot exclude the possibility that in human tumors, the expression of the telomere-associated proteins is a response to, versus a cause of the shortening of the telomeres, although this seems unlikely.

It has previously been shown that the expression of the telomere-associated protein POT1, and possibly TRF2, mRNA decreases in tumor tissue compared with corresponding normal tissue and is associated with disease progression [46]. The relatively small sample size of tumors used for our initial analysis precluded other comparisons, such as the relationships between the expression of telomere-associated proteins and characteristics of the tumor (e.g., grade, size, lymph node status, estrogen receptor status) or patient (e.g., age, outcome). We also did not determine the pattern of expression in matched “normal” or breast tissue. We have previously demonstrated that patient-matched histologically normal breast tissues 1 cm from the tumor margin shares many molecular characteristics with tumor, whereas tissue more distal to the margin (5 cm) or tissue from reduction mammoplasty does not; a phenomenon termed field cancerization [47, 48]. It will be interesting to determine whether the expression of these telomere-associated proteins is also a marker of field-cancerized breast tissue.

By examining the expression of telomere-associated protein mRNAs in human breast tissue and comparing with telomere content, an inverse relationship was demonstrated between the telomere-associated proteins and telomere content. Examination of the promoter regions and in vitro cell line experiments demonstrated that factors that mediate inflammation and stress can affect the expression of the telomere-associated proteins. Further experimentation is required to elucidate the mechanism of action; however, the findings presented herein provide a possible link between inflammation found in cancer and expression of telomere-associated proteins that are required for telomere maintenance and function.
